# In Vitro Antiproliferative Evaluation of Synthetic Meroterpenes Inspired by Marine Natural Products

**DOI:** 10.3390/md17120684

**Published:** 2019-12-05

**Authors:** Concetta Imperatore, Gerardo Della Sala, Marcello Casertano, Paolo Luciano, Anna Aiello, Ilaria Laurenzana, Claudia Piccoli, Marialuisa Menna

**Affiliations:** 1 The NeaNat Group, Department of Pharmacy, University of Naples “Federico II”, Via D. Montesano 49, 80131 Napoli, Italy; cimperat@unina.it (C.I.); marcello.casertano@unina.it (M.C.); pluciano@unina.it (P.L.); aiello@unina.it (A.A.); 2 Laboratory of Pre-Clinical and Translational Research, IRCCS-CROB, Referral Cancer Center of Basilicata, 85028 Rionero in Vulture, Italy; gerardo.dellasala@crob.it (G.D.S.); ilaria.laurenzana@crob.it (I.L.); claudia.piccoli@unifg.it (C.P.); 3 Department of Clinical and Experimental Medicine, University of Foggia, via L. Pinto c/o OO.RR., 71100 Foggia, Italy

**Keywords:** organic synthesis, meroterpenoids, thiazinoquinones, antiproliferative activity, G0/G1 cell-cycle arrest, cytostatic, solid tumor cell lines

## Abstract

Several marine natural linear prenylquinones/hydroquinones have been identified as anticancer and antimutagenic agents. Structure-activity relationship studies on natural compounds and their synthetic analogs demonstrated that these effects depend on the length of the prenyl side chain and on the type and position of the substituent groups in the quinone moiety. Aiming to broaden the knowledge of the underlying mechanism of the antiproliferative effect of these prenylated compounds, herein we report the synthesis of two quinones **4** and **5** and of their corresponding dioxothiazine fused quinones **6** and **7** inspired to the marine natural product aplidinone A (**1**), a geranylquinone featuring the 1,1-dioxo-1,4-thiazine ring isolated from the ascidian *Aplidium conicum*. The potential effects on viability and proliferation in three different human cancer cell lines, breast adenocarcinoma (MCF-7), pancreas adenocarcinoma (Bx-PC3) and bone osteosarcoma (MG-63), were investigated. The methoxylated geranylquinone **5** exerted the highest antiproliferative effect exhibiting a comparable toxicity in all three cell lines analyzed. Interestingly, a deeper investigation has highlighted a cytostatic effect of quinone **5** referable to a G0/G1 cell-cycle arrest in BxPC-3 cells after 24 h treatment.

## 1. Introduction

Chemotherapy represents the most applied strategy for cancer treatment; therefore, development of novel and improved antitumor compounds has become mandatory.

The sustainable exploitation of marine natural products as starting leads is a precious and still untapped resource. Many classes of natural molecules have been conceived by nature to play specific roles in cell processes through selective interactions with key cellular targets. In this frame, quinones represent a clinically relevant class of chemotherapeutic agents with antitumor activity already described in several cell lines [1]. The most prominent chemical feature of these compounds, both natural and synthetic derivatives, is the ability to undergo redox cycling to generate reactive oxygen species (ROS), responsible for significant cell damage. In this class of compounds, a heterogeneous group of prenylated structures formed through the mixed terpenoid-polyketide biosynthetic pathway, stands out for their potential anticancer activities [2,3]. This group of molecules, commonly named meroterpenoids, are broadly widespread in both terrestrial plants, insects, fungi, lichens and marine organisms [4], being involved in electron transport processes and into photosynthesis [5]. Among marine ascidians, these compounds have been found almost exclusively in species belonging to the genus *Aplidium*. Since the first report of the presence of geranylhydroquinone in an *Aplidium* sp., a number of structurally diverse meroterpenes have been isolated from several *Aplidium* species. Among them, a wide variety of often very complex molecules, originating from intra- and intermolecular cyclizations and/or rearrangements of the terpene chains to give unique polycyclic or macrocyclic structures, have been discovered [2,5,6]. In the course of our ongoing research program aimed at the search and characterization of new drug candidates of marine origin [7,8,9,10,11], a large group of new meroterpenes with different polycyclic skeletons but all featuring an unusual 1,1-dioxo-1,4-thiazine ring fused with the quinone moiety, i.e., aplidinones A and B, thiaplidiaquinones and conithiaquinones, were isolated from samples of *Aplidium conicum* [12,13,14]. The valuable antitumor activity shown by these compounds prompted us to further investigate the chemical and pharmacological features of this class of compounds [15]. For this purpose, several synthetic analogs of aplidinone A (**1**), featuring a methoxyl group and a monoprenyl alkyl chain linked at the thiazinoquinones scaffold, have been synthetized, in which the geranyl chain is replaced by other alkyl chains [16,17,18,19]. This synthetic chemical library along with the natural metabolite was subjected to cytotoxicity assays and preliminary structure-activity relationships (SAR) studies. This approach allowed us to define that the cytotoxic effects depend on the nature and the length of side chain linked to the benzoquinone ring and, mainly, on its position respect to the dioxothiazine ring.

**Figure 1:**
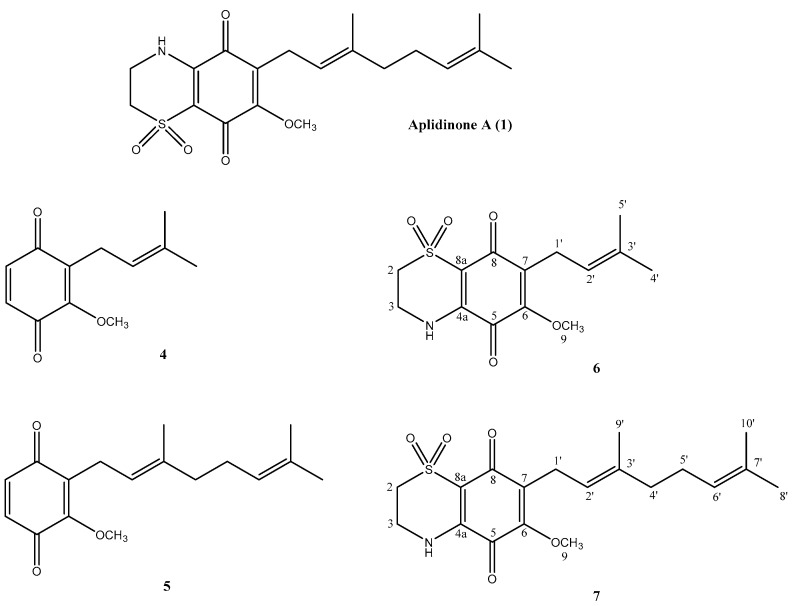
Structures of aplidinone A (**1**) and of the synthetic derivatives **4**–**7**.

In order to expand the chemical library and more clearly establish the role of the thiazine ring and of both length and shape of the alkyl side chain on the cytotoxicity, we have synthesized the two prenylated quinones **4** and **5** and we have then converted them into the corresponding thiazinoquinones **6** and **7** (Figure 1). We have then explored their potential effects on viability and proliferation in three different human cancer cell lines, namely MCF-7 (breast adenocarcinoma), Bx-PC3 (pancreas adenocarcinoma), and MG-63 (bone osteosarcoma). We report herein the synthesis, the chemical characterization and the pharmacological profile of compounds **4**–**7**.

## 2. Results and Discussion

### 2.1. Chemistry

The prenylquinones **4** and **5** as well as the relevant thiazinoquinone derivatives **6** and **7** were synthesized using a synthetic protocol previously designed and developed in order to easily produce and enlarge the chemodiversity within the thiazinoquinones library [16,17,18].

**Scheme 1:**
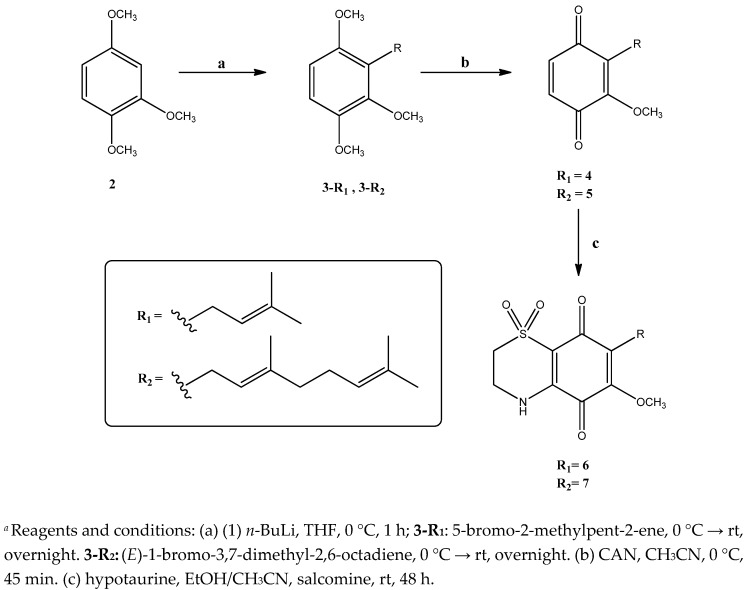
Synthesis of compounds **4**–**7**.

In detail, as reported in Scheme 1, the commercially available 1,2,4-trimethoxybenzene (**2**) has been chosen as the starting material. In the first step, compound **2** was treated with *n*-BuLi in THF solution; 5-bromo-2-methylpent-2-ene or (*E*)-1-bromo-3,7-dimethyl-2,6-octadiene were then added, keeping the relevant mixtures at room temperature overnight. Under these conditions, the monoalkylated products, compounds **3**-**R_1_** and **3**-**R_2_**, were obtained with very high yield, following that the introduction of the alkyl residue on 1,2,4-trimethoxy benzene resulted highly selective in the 3-position. Subsequently, each compound **3**-**R_1_** and **3**-**R_2_** was subjected to an oxidation reaction with cerium ammonium nitrate (CAN) providing the quinones **4** (83%) and **5** (76%), respectively. Previously reported attempts to produce selectively 3-monoprenylated quinones afforded mixtures of monoprenylated compounds together with polyprenylated products [20]. Compound **5** itself had been previously obtained through this different synthetic pathway with an overall low yield (≈ 45%). Our procedure afforded monoprenylated quinone **5** with a total yield of 74%, thus resulting in a great improvement over previous synthesis, and the absence of side products, especially of monoalkylated quinones in different position. Afterwards, the thiazinoquinone-bicyclic system of **6** and **7** was built up by condensation of the quinone ring of compounds **4** and **5** with hypotaurine using salcomine as catalyst. Contrary to what previously occurred during the formation of thiazinoquinone scaffold in other unsymmetrical methoxy-quinones [16,17,18,19], the addition of hypotaurine to prenylbenzoquinones **4** and **5** led to the formation of only one of the two possible regioisomers (Scheme 1). This highly regioselective outcome could be explained by considering the greater steric hindrance of the prenylated side chains of compounds **4** and **5** when compared to other quinones featuring smaller and/or less flexible side chains [16,17,18,19]. 

The above-reported synthetic strategy is particularly advantageous because it starts from low-cost and commercially available reagents allowing to obtaining several dioxothiazinoquinones in few steps and in very good yields. High purity, more than 99.8%, was easily obtained for quinones **4**–**5** as well as for thiazinoquinones **6**–**7** by HPLC; each compound was fully characterized by spectroscopic means (Table 1).

Structures of compounds **4** and **5** were easily defined similar to the spectroscopic resonances (^1^H and ^13^C NMR) of the compounds already reported in literature [20]. HRESI-MS data of compounds **6** and **7** indicated that the two compounds had the molecular formulas C_14_H_17_O_5_NS and C_19_H_25_O_5_NS, respectively. Analysis of 1D and 2D NMR spectral data of **6** and **7** (CDCl_3_) allowed the assignment of all ^1^H and ^13^C NMR signals (Table 1) confirming the whole structure of thiazinoquinones **6** and **7**, except for the regiochemistry of the 1,1-dioxo-1,4-thiazine ring. Nevertheless, the close correlation between the experimental NMR signals of bicyclic skeleton of compounds **6** and **7** with ^13^C chemical shift values previously calculated by theoretical means [14,19] for strictly correlated structures was in agreement with the regiochemistry depicted in Figure 1.

### 2.2. In Vitro Evaluation of Antiproliferative Activity of Quinones ***4***–***7*** in Cancer Cell Lines

Aiming to assess antiproliferative activity and structure-activity relationships of synthetic quinones **4**–**7** in solid tumor models, potential growth inhibitory effects were evaluated in three different human cancer cell lines, namely MCF-7 (breast adenocarcinoma), Bx-PC3 (pancreas adenocarcinoma), and MG-63 (bone osteosarcoma). The cell viability was monitored by a real-time cell analyzer based upon impedance measurements of cells growing on microelectronic sensors (xCELLigence system-ACEA Biosciences, San Diego, CA, USA). Drug-induced cell growth inhibition prompts alterations of electronic impedance, which are expressed as cell index (CI), a unit-less parameter indicative of cell number and morphology. 

**Figure 2:**
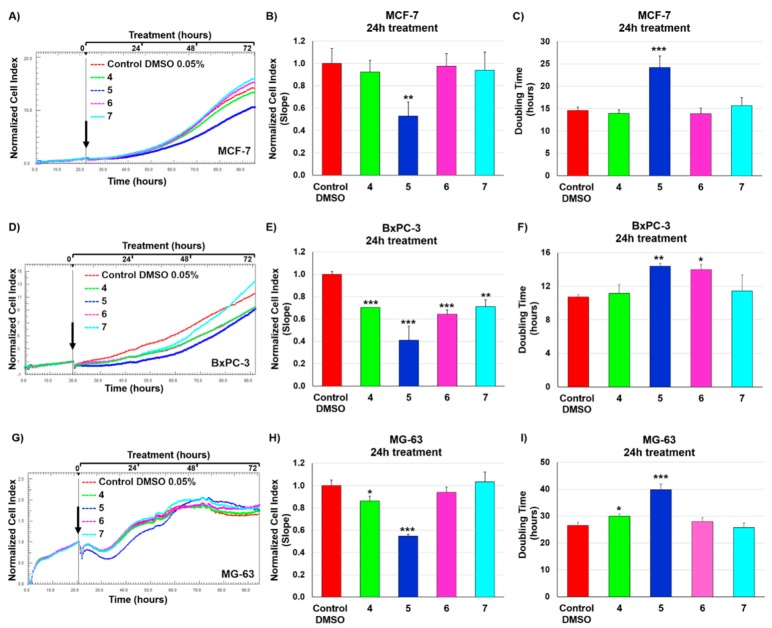
Real time monitoring of cancer cell growth after exposure to quinones **4**–**7** (10 µM) and DMSO vehicle (0.05%) using the xCELLigence System Real-Time Cell Analyzer. (**A**,**D**,**G**) Normalized cell index (NCI) traces of MCF7 (**A**), BxPC-3 (**D**), and MG-63 (**G**) cells exposed to compounds **4**–**7** and DMSO vehicle for 72 h. Black arrow shows the starting point of drug treatment. Each cell index value was normalized just before treatment. (**B**,**E**,**H**) NCI variations of MCF-7 (**B**), BxPC-3 (**E**), and MG-63 (**H**) cells after 24 h exposure to compounds **4**–**7** (10 μM) and 0.05% DMSO vehicle. Antiproliferative effects are reported as slope of NCI to describe the changing rate of growth curves after drug treatment. NCI slope values are relative to controls treated with DMSO vehicle. (**C**,**F**,**I**) Doubling times of NCI of MCF-7 (**C**), BxPC-3 (**F**), and MG-63 (**I**) cells after 24h incubation with 10 μM of quinones **4**–**7** and 0.05% DMSO. Data are presented as mean ± SD; *n* = 3. Statistical significances are referred to the DMSO control. * *p* < 0.05; ** *p* < 0.01; *** *p* < 0.0001.

Quinones **4**–**7** were initially tested individually at a single dose exposure (10 µM) for 72 h. Real-time monitoring of cell proliferation (Figure 2) unveiled that a) Bx-PC3 cells were the most sensitive cell line and b) quinones were more effective during the first 24 h, which was selected as time point for our following investigations. 

Bx-PC3 cells experienced delayed proliferation after exposure to compounds **4**, **6**, and **7**, which were shown to elicit a significant, moderate reduction (approximately 30%) in the slope of the growth curve as compared to the control. On the other hand, MCF-7 and MG-63 cell growth was basically unaffected or slightly delayed after treatment with **4**, **6**, and **7** (Figure 2A–C,G–I). Notably, compound **5** exerted the highest antiproliferative effect and exhibited a comparable toxicity in all three cell lines, as inducing a substantial decrease of cell index (within the range of 50–60%) and a significant increase in cell doubling time.

In the light of these findings, investigation of structure-activity relationships revealed key structural motifs for maintaining growth inhibitory properties of the molecules under examination. The prenyl chain length appears to be crucial to keep a strong bioactivity profile as addition of a second prenyl unit in **5** improves antiproliferative effects in the tested cell lines as compared to **4**. Moreover, the presence of a fused thiazine ring weakens cell viability effects. Indeed, compound **5** resulted to be more active than the relevant thiazinoquinone derivative **7**, where C8a and C4a electrophilic sites are embedded in the bicyclic structure of the molecule and, therefore, are not available for potential covalent binding to specific antitumor targets (e.g., ubiquitin-proteasome pathway) [21].

**Figure 3:**
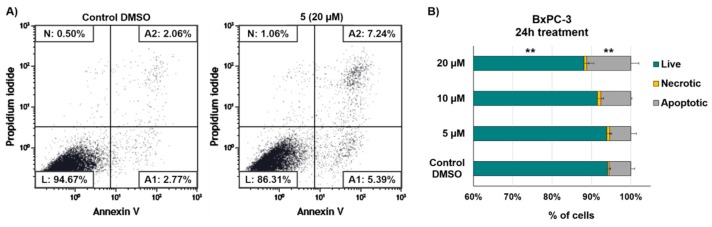
Flow cytometric detection of apoptosis and necrosis with Annexin-V-fluorescein isothiocyanate (FITC) and propidium iodide staining in BxPC3 cells after 24 h exposure to different concentrations of quinone **5** and DMSO vehicle. (**A**) Dot plots show a single representative experiment. Abbreviations: L, live cells; A1, early apoptotic cells; A2, late apoptotic cells; N, necrotic cells. (**B**) Relative amount of live, necrotic, and apoptotic cells after 24 h treatment with different concentrations of **5** (5, 10, and 20 µM) and DMSO vehicle. Percent of apoptotic cells was obtained from the sum of early and late apoptosis. Data are presented as mean ± SD; *n* = 3. Statistical significances are referred to the DMSO control. ** *p* < 0.01.

To evaluate the underlying mechanism of antiproliferative effect of compound **5**, we examined whether it elicited increased apoptotic cell death in BxPC-3 cancer cells by the annexin V-FITC/PI assay (Figure 3). After 24 h incubation with quinone **5** at concentrations of 5, 10 and 20 µM, pancreatic tumor cells exhibited a slight, although significant, increase of early and late apoptotic cells only at the highest dose tested (20 µM), as compared to the control cells treated with DMSO vehicle. The relatively low number of apoptotic cells (approximately 11% at the highest concentration), suggested a cytostatic rather than a cytotoxic effect shown by the compound **5**.

**Figure 4:**
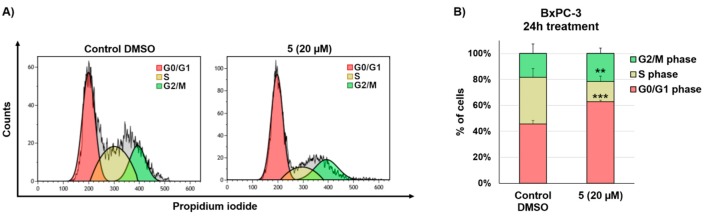
Cell cycle analysis through PI staining and flow cytometry of BxPC-3 cells after 24 h treatment with 20 µM compound **5** and DMSO vehicle. (**A**) Cell cycle histogram plots show a single representative experiment. (**B**) Changes in cell cycle distribution of BxPC-3 cells treated with quinone **5** (20 µM) for 24 h. Data are presented as mean ± SD; *n* = 4. Statistical significances are referred to the DMSO control. ** *p* < 0.01, *** *p* < 0.0001.

As drug-mediated growth inhibition observed during real time cell analysis did not correlate with enhanced apoptosis, we next performed cell cycle analysis by flow cytometry based on PI staining (Figure 4). A typical G0/G1 cell-cycle arrest was observed in BxPC-3 cells after 24 h treatment with quinone **5** (20 µM), shown by a significant increase in G0/G1 phase cells shifting from 45.7% to 62.8% (*p* < 0.0001).

## 3. Materials and Methods 

Commercial reagents: Sigma–Aldrich (Saint Louis, MO, USA). Solvents: Carlo Erba (Pomezia, Rome, Italy). TLC: Silica Gel 60 F254 (plates 5 × 20, 0.25 mm) Merck (Kenilworth, NJ, USA). Preparative TLC: Silica Gel 60 F254 plates (20 × 20, 2 mm). Spots revealed by UV lamp then by spraying with 2 N sulfuric acid and heating at 120 °C. Anhydrous solvents: Sigma–Aldrich or prepared by distillation according to standard procedures. High-resolution ESI-MS analyses were performed on a Thermo LTQ Orbitrap XL mass spectrometer (Thermo-Fisher, San Josè, CA, USA). The spectra were recorded by infusion into the ESI (Thermo-Fisher, San Josè, CA, USA) source dissolving the sample in MeOH. ^1^H (700 MHz and 500 MHz) and ^13^C (125 MHz) NMR spectra were recorded on a Agilent INOVA spectrometer (Agilent Technology, Cernusco sul Naviglio, Italy) equipped with a ^13^C enhanced HCN Cold Probe; chemical shifts were referenced to the residual solvent signal (CDCl_3_: δ_H_ = 7.26, δ_C_ = 77.0). For an accurate measurement of the coupling constants, the one-dimensional ^1^H NMR spectra were transformed at 64 K points (digital resolution: 0.09 Hz). Homonuclear (^1^H-^1^H) and heteronuclear (^1^H-^13^C) connectivities were determined by COSY and HSQC experiments, respectively. Two and three bond ^1^H-^13^C connectivities were determined by gradient 2D HMBC experiments optimized for a ^2,3^*J* of 8 Hz. ^3^*J*_H-H_ values were extracted from 1D ^1^H NMR. High performance liquid chromatography (HPLC) separations were achieved on a Shimadzu LC-10AT (Shimadzu, Milan, Italy) apparatus equipped with a Knauer K-2301 (LabService Analytica s.r.l., Anzola dell’Emilia, Italy) refractive index detector.

### 3.1. Chemistry

#### 3.1.1. Synthesis of 1,2,4-trimethoxy-3-(3-methylbut-2-en-1-yl)benzene (**3**-**R_1_**) and (E)-2-(3,7-dimethylocta-2,6-dien-1-yl)-1,3,4-trimethoxybenzene (**3**-**R_2_**) 

A quantity of 500 µL of 1,2,4-trimethoxybenzene (**2**) (3.4 mmol) was solubilized in 15 mL of THF dry and 2.5 mL of *n*-BuLi (4 mmol) were added to the mixture which was stirred under Argon atmosphere for 1 h at 0 °C. Subsequently, 4 mmol of 3,3-dimethylallyl bromide (470 µL, 4 mmol) for compound **3**-**R_1_** and (*E*)-1-bromo-3,7-dimethyl-2,6-octadiene (800 µL, 4 mmol) for compound **3**-**R_2_** were added respectively keeping the relevant mixture under magnetic stirring at room temperature overnight. After 12 h, both obtained mixtures were quenched with an aqueous solution of sodium chloride (30 mL) and extracted two times with diethyl ether (50 mL). The organic layers were dried over anhydrous sodium sulfate and, concentrated in vacuo to afford **3**-**R_1_** (787 mg, 98%) and **3**-**R_2_** (1 g, 97%) sufficiently pure to the following reaction step. 

1,2,4-trimethoxy-3-(3-methylbut-2-en-1-yl)benzene (**3**-**R_1_**): dark yellow oil; HRESIMS *m*/*z* 259.1307 [M + Na]^+^ (calcd. for C_14_H_20_O_3_Na 259.1305). ^1^H NMR (CDCl_3_, 500 MHz): *δ* 6.72 (1H, d, *J* = 8.9 Hz), 6.57 (1H, d, *J* = 8.9 Hz), 5.23 (1H, t), 3.84 (3H, s), 3.83 (3H, s), 3.79 (3H, s), 3.39 (2H, d, *J* = 7.0 Hz), 1.81 (3H, s), 1.69 (3H, s). ^13^C NMR (CDCl_3_, 125 MHz): *δ* 154.6, 150.5, 149.7, 133.8, 127.5, 125.3, 112.4, 108.1, 63.3, 58.8, 58.6, 28.4, 25.8, 20.4. ^1^H, ^13^C and HRESIMS spectra are reported in Supporting Information (Figures S1–S3).

(*E*)-2-(3,7-dimethylocta-2,6-dien-1-yl)-1,3,4-trimethoxybenzene (**3**-**R_2_**): dark yellow oil; HRESIMS *m*/*z* 327.1942 [M + Na]^+^ (calcd. for C_19_H_28_O_3_Na: 327.1931).^1^H NMR (CDCl_3_, 500 MHz): *δ* 6.73 (1H, d, *J* = 8.9 Hz), 6.58 (1H, d, *J* = 8.9 Hz), 5.24 (1H, t), 5.10 (1H, t), 3.84 (6H, s), 3.80 (3H, s), 3.41 (2H, d, *J* = 6.9 Hz), 2.08 (2H, dd, *J* = 7.5, 6.9 Hz), 2.00 (2H, m), 1.81 (3H, s), 1.67 (3H, s), 1.60 (3H, s). ^13^C NMR (CDCl_3_, 125 MHz): *δ* 154.9, 150.6, 149.9, 137.3, 133.6, 127.8, 127.3, 125.8, 112.3, 108.3, 63.4, 58.9, 58.5, 42.5, 29.4, 28.2, 25.6, 20.4, 18.8. ^1^H, ^13^C and HRESIMS spectra are reported in Supporting Information (Figures S4–S6).

#### 3.1.2. Synthesis of 2-methoxy-3-(3-methylbut-2-en-1-yl)cyclohexa-2,5-diene-1,4-dione (**4**)

A portion of compound **3**-**R_1_** (310 mg, 1.3 mmol) was dissolved in 50 mL of acetonitrile (ACN) at 0 °C. A solution of 2.9 g (5.2 mmol) of ammonium cerium nitrate (CAN) in 9 mL of water was prepared and added dropwise stirring the achieved mixture for 45 min at 0 °C. The end of reaction was monitored by TLC with an eluent system chloroform/EtOAc 7:3 before diluting the orange liquid with 100 mL of water and then extracted with diethyl ether (2 × 100 mL). The consequent organic phase was washed with brine, dried over anhydrous Na_2_SO_4_, filtered and the solvent removed in vacuo. The mixture was chromatographed by HPLC on SiO_2_ column (Luna 3 µm, 150 × 4.6 mm, flow rate 1 mL/min) with a mobile phase hexane/EtOAc 9:1 (*v*/*v*) affording the quinone **4** as a pure compound (221 mg, 83%, t_R_ 4.9 min).

2-methoxy-3-(3-methylbut-2-en-1-yl)cyclohexa-2,5-diene-1,4-dione (**4**): yellow powder, HRESIMS *m*/*z* 229.0839 [M + Na]^+^ (calcd. for C_12_H_14_O_3_Na: 229.0835). ^1^H NMR (CDCl_3_, 500 MHz): *δ* 6.67 (1H, d, *J* = 9.5 Hz), 6.58 (1H, d, *J* = 9.5 Hz), 5.04 (1H, t, *J* = 6.9 Hz), 4.01 (3H, s), 3.13 (2H, d, *J* = 7.3 Hz), 1.73 (3H, s), 1.66 (3H, s). ^13^C NMR (CDCl_3_, 125 MHz): *δ* 187.4, 183.4, 155.0, 136.8, 136.1, 134.4, 131.9, 119.4, 60.7, 25.2, 22.3, 17.5. ^1^H, ^13^C and HRESIMS spectra are reported in Supporting Information (Figures S7–S9).

#### 3.1.3. Synthesis of (E)-2-(3,7-dimethylocta-2,6-dien-1-yl)-3-methoxycyclohexa-2,5-diene-1,4-dione (**5**)

A quantity of 130 mg (0.43 mmol) of compound **3**-**R_2_** was dissolved in 20 mL of ACN and an aqueous solution of CAN (938 mg, 1.7 mmol in 5 mL of water) was added dropwise to the mixture in a cold bath at 0 °C. The orange mixture was kept under magnetic stirring for 45 min at the above-mentioned temperature monitoring the reaction progress by TLC (chloroform/EtOAc 7:3). After this time, the solution was diluted with 100 mL of cold water and subjected to an extraction twice with 80 mL of diethyl ether. The collected organic layer was washed with a saturated solution of NaCl, dried over anhydrous Na_2_SO_4_, filtered and the solvent removal was realized under reduced pressure. The pure quinone **5** (80 mg, 76%) was obtained by HPLC purification of the crude residue on silica gel column (Luna 3 µm, 150 × 4.6mm, flow rate 1 mL/min) and hexane/EtOAc 98:2 (*v*/*v*) as mobile phase (t_R_ 20.4 min). 

(*E*)-2-(3,7-dimethylocta-2,6-dien-1-yl)-3-methoxycyclohexa-2,5-diene-1,4-dione (**5**): yellow powder, HRESIMS *m*/*z* 275.1645 [M + H]^+^ (calcd. for C_17_H_23_O_3_: 275.1642). ^1^H NMR (CDCl_3_, 500 MHz): *δ* 6.67 (1H, d, *J* = 9.5 Hz), 6.59 (1H, d, *J* = 9.5 Hz), 5.05 (2H, m, overlapped), 4.02 (3H, s), 3.16 (2H, d, *J* = 7.3 Hz), 2.05 (2H, m), 1.96 (2H, m), 1.73 (3H, s), 1.65 (3H, s), 1.58 (3H, s). ^13^C NMR (CDCl_3_, 125 MHz): *δ* 187.8, 183.7, 155.2, 137.1, 136.6, 134.4, 132.2, 131.3, 123.9, 119.6, 60.8, 39.5, 26.5, 25.4, 22.4, 17.7, 16.0. ^1^H, ^13^C and HRESIMS spectra are reported in Supporting Information (Figures S10–S12).

#### 3.1.4. Synthesis of 6-methoxy-7-(3-methylbut-2-en-1-yl)-3,4-dihydro-2H-benzo[b][1,4]thiazine-5,8-dione-1,1-dioxide (**6**) and (*E*)-7-(3,7-dimethylocta-2,6-dien-1-yl)-6-methoxy-3,4-dihydro-2H-benzo[b][1,4]thiazine-5,8-dione-1,1-dioxide (**7**)

The thiazinoquinone compounds, **6** and **7**, have been synthesized by the coupling of the respective quinone ring, **4** and **5**, with hypotaurine. For this purpose, 137 mg (0.67 mmol) of **4** were solubilized in 15 mL of a mixture ACN/EtOH 1:1 (*v*/*v*) while 14 mg (0.049 mmol) of **5** were solubilized in 5 mL of the same mixture. A water solution of hypotaurine (66.4 mg, 0.67 mmol in 3 mL for the synthesis of **6** and 6.0 mg, 0.049 mmol in 500 µL for the synthesis of **7**) was added dropwise to relative quinone. Salcomine as catalyst was added in portion and the mixtures were kept under stirring at room temperature for 48 h. After observing a color change from yellow to orange, TLC eluted with a mixture chloroform/EtOAc 7:3 allowed to control the end of the condensation before removing the solvent at rotavapor. The residues were dissolved in water and the mixtures were extracted with diethyl ether (3 × 60 mL). The organic phases were washed with brine, dried, filtered, and concentrated in vacuo. ^1^H NMR spectra recorded for the two crude residues showed the presence in both cases of a single regioisomer which have been purified by HPLC on silica gel (Luna 3 µm column, 150 × 4.6mm, flow rate 1 mL/min) with a mobile phase hexane/EtOAc 1:1 (*v*/*v*) giving compounds **6** (190 mg, 92%) and **7** (15,1 mg, 81%), respectively.

6-methoxy-7-(3-methylbut-2-en-1-yl)-3,4-dihydro-2H-benzo[b][1,4]thiazine-5,8-dione-1,1-dioxide (**6**): slight orange powder; HRESIMS *m*/*z* 312.0909 [M + H]^+^ (calcd. for C_14_H_18_O_5_NS: 312.0901); *m*/*z* 334.0728 [M + Na]^+^ (calcd. for C_14_H_17_O_5_NSNa: 334.0720). ^1^H and ^13^C NMR data are reported in Table 1; NMR spectra are reported in Supporting Information (Figures S13–S15). HRESIMS spectrum is reported in Supporting Information (Figure S16).

(*E*)-7-(3,7-dimethylocta-2,6-dien-1-yl)-6-methoxy-3,4-dihydro-2H-benzo[b][1,4]thiazine-5,8-dione-1,1-dioxide (**7**): slight orange powder; HRESIMS *m*/*z* 380.1519 [M + H]^+^ (calcd. for C_19_H_26_O_5_NS: 380.1526; HRESIMS *m*/*z* 402.1337 [M + Na]^+^ (calcd. for C_19_H_25_O_5_NSNa: 402.1346). ^1^H and ^13^C NMR data are reported in Table 1; NMR spectra are reported in Supporting Information (Figures S17–S19). HRESIMS spectrum is reported in Supporting Information (Figure S20).

### 3.2. In Vitro Evaluation of Antiproliferative Activity of Compounds ***4***–***7*** in Cancer Cell Lines

#### 3.2.1. Cell Culture

MCF-7, BxPC-3 and MG-63 cells were purchased from American Type Culture Collection (ATCC, Manassas, VA, USA). MCF-7 and MG-63 cells were cultured in DMEM medium, while BxPC-3 in RPMI medium, at 37 °C in a 5% CO_2_ humidified atmosphere. DMEM and RPMI media were supplemented with 10% fetal bovine serum, penicillin–streptomycin (100 U/mL), and 2 mM l-glutamine. Cell morphology was monitored by using an inverted optical microscope. Cells were detached with 0.05% trypsin-EDTA to perform in vitro assays.

#### 3.2.2. xCELLigence Assays

Antiproliferative assays were performed by using the xCELLigence System Real-Time Cell Analyzer (ACEA Biosciences, San Diego, CA, USA), as previously described [22]. MCF-7 cells were seeded at a cell density of 3000 cells/well, BxPC-3 at a cell density of 2500 cells/well, and MG-63 cells at a cell density of 4000 cells/well. Approximately 24 h after seeding, cancer cells were treated with 10 μM of compounds **4**–**7** and 0.05% DMSO vehicle for 72 h. 

For data analysis, cell index (CI) values were normalized just before drug treatment to have normalized cell index (NCI) values. Normalized cell index was calculated as follows: NCI = CI _end of treatment_/CI _normalization time_. Real-time NCI proliferation curves were generated through the Real-Time Cell Analyzer (RTCA)-integrated software (Version 2.0.0.1301, ACEA Biosciences, San Diego, CA, USA). Growth inhibitory effects of compounds **4**–**7** are expressed either as cell index slopes relative to controls treated with DMSO vehicle or as cell doubling times. Cell index slopes and doubling times were calculated using the RTCA-integrated software within a 24-h time window. 

#### 3.2.3. Apoptosis Assay and Cell Cycle Analysis

After treatment with different concentrations (5, 10, and 20 µM) of compound **5**, BxPC-3 cells were stained with annexin-V-fluorescein isothiocyanate (FITC) and propidium iodide, using the FITC Annexin V Apoptosis Detection kit I (Becton Dickinson, BD, Franklin, NJ, USA) for flow cytometric detection of apoptotic and necrotic cells. Samples were prepared according to manufacturer’s protocol and three independent experiments were carried out. 

For cell cycle analysis of BxPC-3 cells treated with 20 µM of quinone **5** for 24 h, pancreatic cancer cells were permeabilized with 70% cold ethanol for 1 h and stained for 30 min with a solution containing 50 µg/mL propidium iodide (Sigma Aldrich, St. Louis, MO, USA) and 10 µg/mL RNase A (EuroClone S.p.a., Pero, MI, Italy) in calcium and magnesium-free PBS. Four independent experiments were carried out. All samples were acquired by NAVIOS flow cytometer and analysed by Kaluza software (Beckman Coulter). 10,000 events were acquired for each sample.

#### 3.2.4. Statistical Analysis

Data represent the mean (±standard deviation, SD) of at least three independent experiments. The one-way analysis of variance (ANOVA) method was applied to compare means of more than two groups and Dunnett’s method was used as post-hoc test to compare multiple groups versus a control group. Two-group comparisons were performed using Student’s *t*-test. *p*-values < 0.05 were considered to be statistically significant. Statistical analysis was performed using the GraphPad Prism Software Version 5 (GraphPad Software Inc., San Diego, CA, USA).

## 4. Conclusions

The synthesis of the prenylated compounds **4**–**7,** inspired by the marine thiazinoquinone aplidinone A, has been performed through a versatile synthetic protocol, and it represents an example of a successful strategy. In addition, our synthetic procedure resulted in a considerable improvement of the overall yield of **5** with respect to previously reported synthesis [20]. Moreover, the nucleophilic addition of hypotaurine to quinone ring of **4** and **5** to give the thiazinoquinone **6** and **7**, respectively, was completely regioselective with respect to condensation with other quinones featuring smaller and /or less flexible side chains [16,17,18,19] indicating a key role of the side chain in the condensation reaction. To assess the anticancer potential and structure-activity relationships of compounds **4**–**7**, we evaluated their effects on cell viability of MCF-7, Bx-PC3, and MG-63 cell lines. The biological assays demonstrated the quinone **5** as the most relevant compound in the series exhibiting a similar extent of toxicity against three different tumor models. Finally, compound **5** exerted cytostatic activity through induction of cell cycle arrest, resulting in a significant segregation of cells into G0/G1 phase at concentration of 20 μM. 

## Figures and Tables

**Table 1:** ^1^H (700 MHz) and ^13^C NMR (125 MHz) spectroscopic data ^a^ of compounds **6** and **7** in CDCl_3_.

	6 ^a^		7 ^a^	
Pos.	δ_C_	δ_H,_ mult. (*J* in Hz)	δ_C_	δ_H,_ mult. (*J* in Hz)
**1**	-	-	-	-
**2**	49.0	3.29, m	49.0	3.28, m
**3**	39.9	4.04, m	39.7	4.04, m
**4**	-	-	-	-
**4a**	142.9	-	143.2	-
**5**	176.8	-	176.5	-
**6**	152.7	-	153.1	-
**7**	136.9	-	137.0	-
**8**	178.1	-	178.8	-
**8a**	110.2	-	109.8	-
**9**	60.8	3.91, s	61.0	3.89, s
**1′**	22.9	3.20, d (7.3)	22.9	3.20, d (5.5)
**2′**	119.1	5.04, t (7.6)	119.5	5.04, m ^b^
**3′**	134.5	-	138.5	-
**4′**	17.8	1.72, s	39.9	1.93, m
**5′**	25.7	1.65, s	26.5	2.02, m
**6′**	-	-	124.5	5.04, m ^b^
**7′**	-	-	131.8	-
**8′**	-	-	17.8	1.56, s
**9′**	-	-	16.9	1.71, s
**10′**	-	-	25.3	1.64, s
**-NH**		6.57, brs	-	6.84, brs

^a 1^H NMR and ^13^C NMR shifts are referenced to CDCl_3_ (δ_H_ = 7.26 ppm and δ_C_ = 77.0 ppm). ^b^ Partially overlapped to other resonances.

## Supplementary Materials

The following are available online at https://www.mdpi.com/1660-3397/17/12/684/s1. Figure S1: ^1^H NMR spectrum in CDCl_3_ (500 MHz) of compound **3**-**R_1_**. Figure S2: ^13^C NMR spectrum in CDCl_3_ (125 MHz) of compound **3**-**R_1_**. Figure S3: HRESIMS spectrum of compound **3**-**R_1_**. Figure S4: ^1^H NMR spectrum in CDCl_3_ (500 MHz) of compound **3**-**R_2_**. Figure S5: ^13^C NMR spectrum in CDCl_3_ (125 MHz) of compound **3**-**R_2_**. Figure S6: HRESIMS spectrum of compound **3**-**R_2_**. Figure S7: ^1^H NMR spectrum in CDCl_3_ (500 MHz) of compound **4**. Figure S8: ^13^C NMR spectrum in CDCl_3_ (125 MHz) of compound **4**. Figure S9: HRESIMS spectrum of compound **4**. Figure S10: ^1^H NMR spectrum in CDCl_3_ (500 MHz) of compound **5**. Figure S11: ^13^C NMR spectrum in CDCl_3_ (125 MHz) of compound **5**. Figure S12: HRESIMS spectrum of compound **5**. Figure S13: ^1^H NMR spectrum in CDCl_3_ (500 MHz) of compound **6**. Figure S14: ^13^C NMR spectrum in CDCl_3_ (125 MHz) of compound **6**. Figure S15: ^1^H-^13^C HMBC spectrum in CDCl_3_ (700 MHz) of compound **6**. Figure S16: HRESIMS spectrum of compound **6**. Figure S17: ^1^H NMR spectrum in CDCl_3_ (500 MHz) of compound **7**. Figure S18: ^13^C NMR spectrum in CDCl_3_ (125 MHz) of compound **7**. Figure S19: ^1^H-^13^C HMBC spectrum in CDCl_3_ (700 MHz) of compound **7**. Figure S20: HRESIMS spectrum of compound **7**. Figure S21: HPLC chromatogram of compound **4**. Figure S22: HPLC chromatogram of compound **5**. Figure S23: HPLC chromatogram of compound **6**. Figure S24: HPLC chromatogram of compound **7**.
