# Supplementary material for: In Vitro Antiproliferative Evaluation of Synthetic Meroterpenes Inspired by Marine Natural Products
Source: Mar Drugs. 2019 Dec 5;17(12):684. doi: 10.3390/md17120684 (PMC6950182; doi:10.3390/md17120684)

In vitro antiproliferative evaluation of synthetic meroterpenes inspired by marine natural products

Concetta Imperatore[1,†], Gerardo Della Sala[2,†], Marcello Casertano[1], Paolo Luciano[1], Anna Aiello[1], Ilaria Laurenzana[2], Claudia Piccoli[2,3], and Marialuisa Menna[1,*]

[1] The NeaNat Group, Department of Pharmacy, University of Naples "Federico II", Via D. Montesano 49, 80131 Napoli, Italy; cimperat@unina.it (C.I.); marcello.casertano@unina.it (M.C.); pluciano@unina.it (P.L.); aiello@unina.it (A.A.)

[2] Laboratory of Pre-Clinical and Translational Research, IRCCS-CROB, Referral Cancer Center of Basilicata, 85028 Rionero in Vulture, Italy; gerardo.dellasala@crob.it (G.D.S.); ilaria.laurenzana@crob.it (I.L.)

[3] Department of Clinical and Experimental Medicine, University of Foggia, via L. Pinto c/o OO.RR., 71100 Foggia, Italy; claudia.piccoli@unifg.it (C.P.)

* Correspondence: mlmenna@unina.it (M.M.); Tel.: +39-081-678518

† These authors contributed equally to this work.

Received: date; Accepted: date; Published: date

TABLE OF CONTENTS

Figure S1. ^{1}H NMR spectrum in $CDCl_3$ (500 MHz) of compound **3-R$_1$** 3
Figure S2. ^{13}C NMR spectrum in $CDCl_3$ (125 MHz) of compound **3-R$_1$** 3
Figure S3. HRESIMS spectrum of compound **3-R$_1$** 4
Figure S4. ^{1}H NMR spectrum in $CDCl_3$ (500 MHz) of compound **3-R$_2$** 4
Figure S5. ^{13}C NMR spectrum in $CDCl_3$ (125 MHz) of compound **3-R$_2$** 5
Figure S6. HRESIMS spectrum of compound **3-R$_2$** 5
Figure S7. ^{1}H NMR spectrum in $CDCl_3$ (500 MHz) of compound **4** 6
Figure S8. ^{13}C NMR spectrum in $CDCl_3$ (125 MHz) of compound **4** 6
Figure S9. HRESIMS spectrum of compound **4** 7
Figure S10. ^{1}H NMR spectrum in $CDCl_3$ (500 MHz) of compound **5** 7
Figure S11. ^{13}C NMR spectrum in $CDCl_3$ (125 MHz) of compound **5** 8
Figure S12. HRESIMS spectrum of compound **5** 8
Figure S13. ^{1}H NMR spectrum in $CDCl_3$ (500 MHz) of compound **6** 9
Figure S14. ^{13}C NMR spectrum in $CDCl_3$ (125 MHz) of compound **6** 9
Figure S15. ^{1}H-^{13}C HMBC spectrum in $CDCl_3$ (700 MHz) of compound **6** 10
Figure S16. HRESIMS spectrum of compound **6** 10
Figure S17. ^{1}H NMR spectrum in $CDCl_3$ (500 MHz) of compound **7** 11
Figure S18. ^{13}C NMR spectrum in $CDCl_3$ (125 MHz) of compound **7** 11
Figure S19. ^{1}H-^{13}C HMBC spectrum in $CDCl_3$ (700 MHz) of compound **7** 12
Figure S20. HRESIMS spectrum of compound **7** 12
Figure S21. HPLC chromatogram of compound **4** 13
Figure S22. HPLC chromatogram of compound **5** 13
Figure S23. HPLC chromatogram of compound **6** 13
Figure S24. HPLC chromatogram of compound **7** 14

Figure S1. ^{1}H NMR spectrum in $CDCl_3$ (500 MHz) of compound **3-R₁**

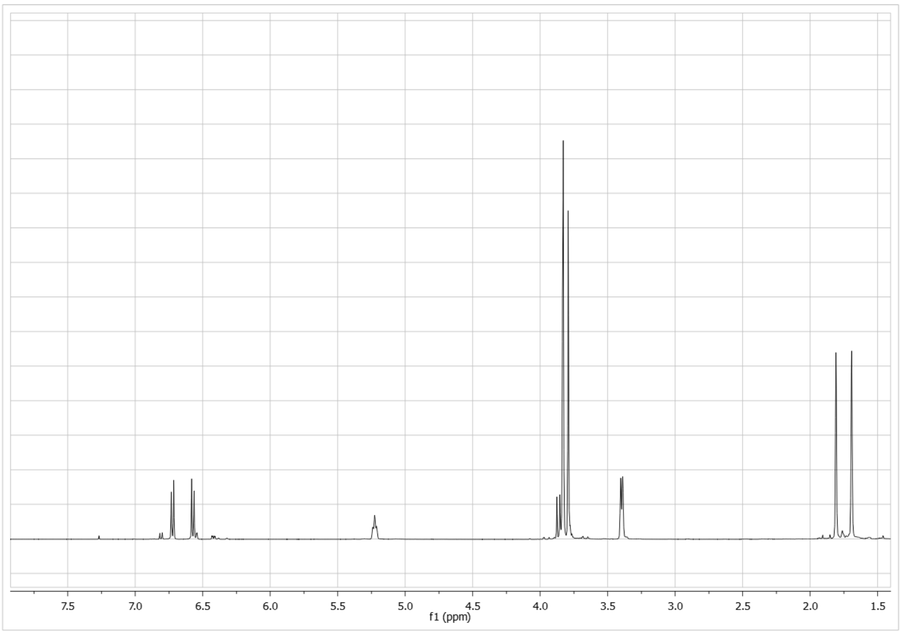

Figure S2. ^{13}C NMR spectrum in $CDCl_3$ (125 MHz) of compound **3-R₁**

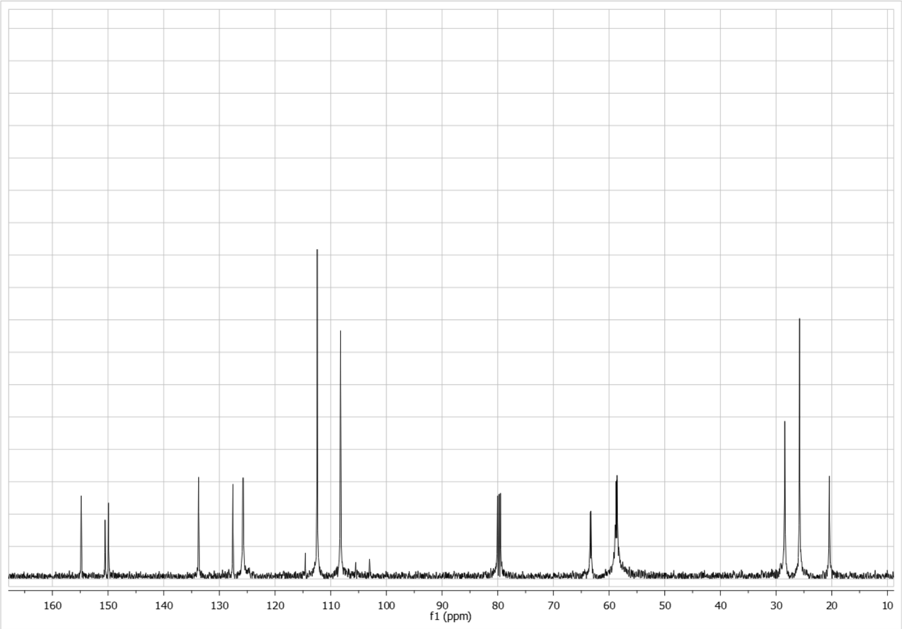

Figure S3. HRESIMS spectrum of compound **3-R**$_1$

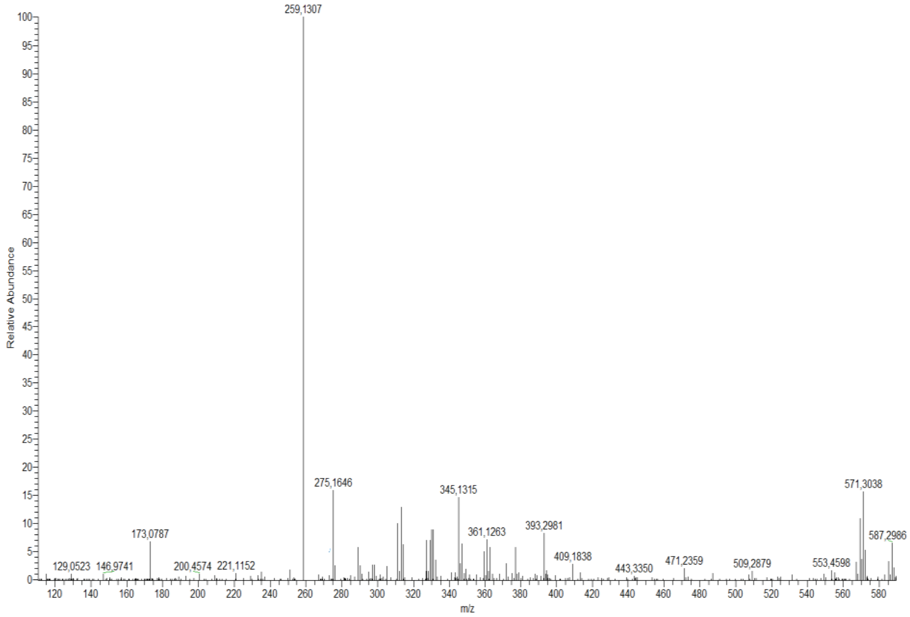

Figure S4. ^{1}H NMR spectrum in $CDCl_3$ (500 MHz) of compound **3-R**$_2$

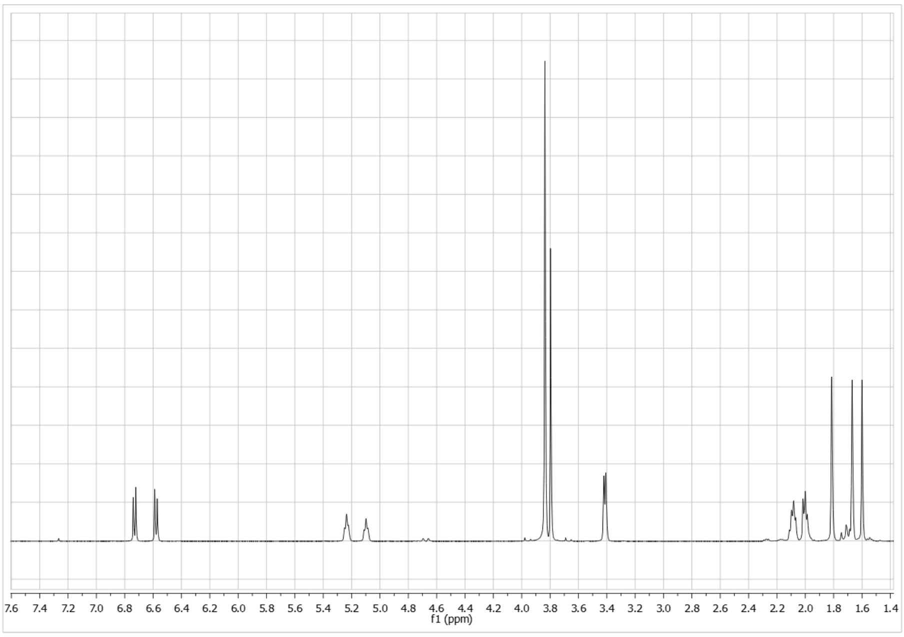

Figure S5. ^{13}C NMR spectrum in $CDCl_3$ (125 MHz) of compound **3-R$_2$**

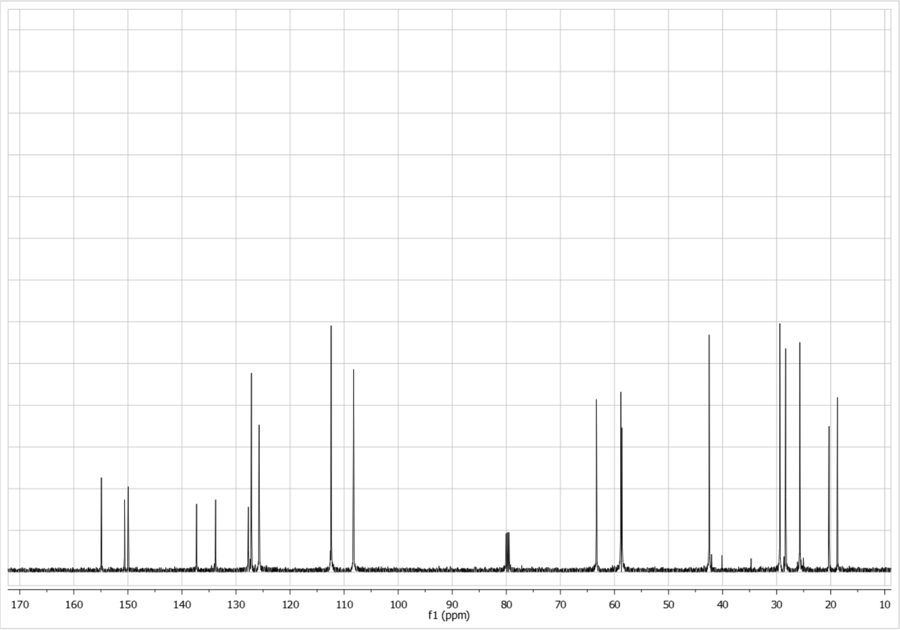

Figure S6. HRESIMS spectrum of compound **3-R$_2$**

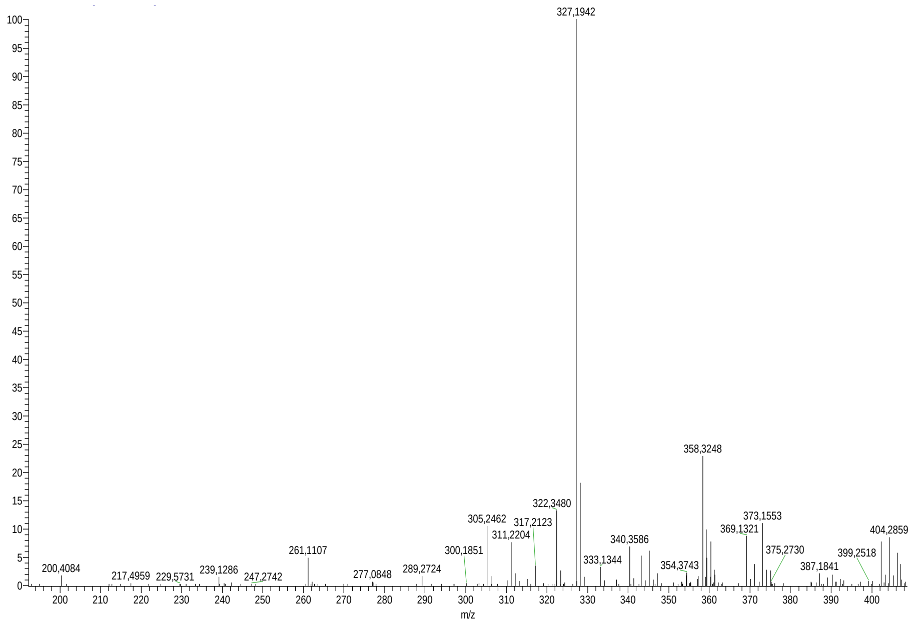

Figure S7. ^{1}H NMR spectrum in $CDCl_3$ (500 MHz) of compound **4**

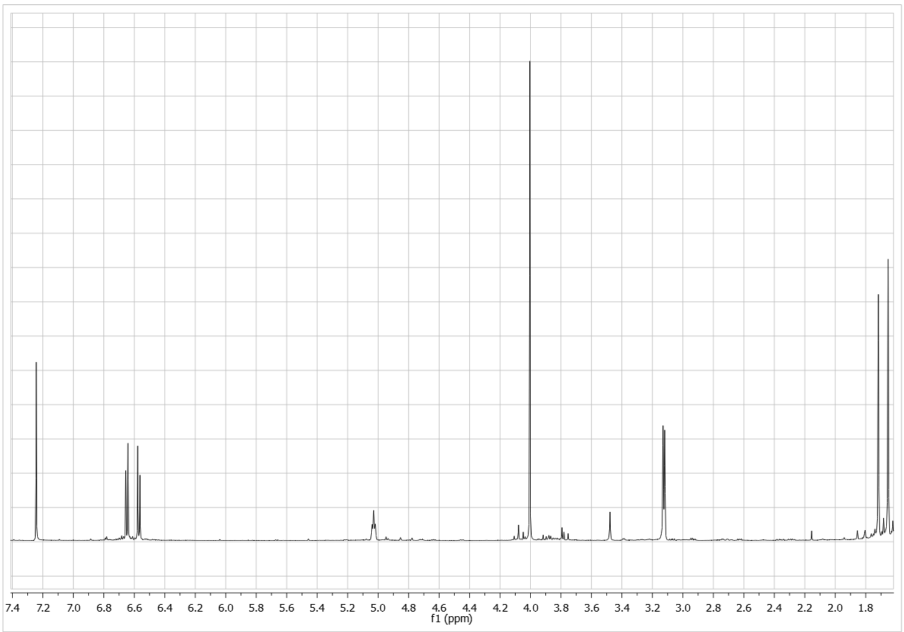

Figure S8. ^{13}C NMR spectrum in $CDCl_3$ (125 MHz) of compound **4**

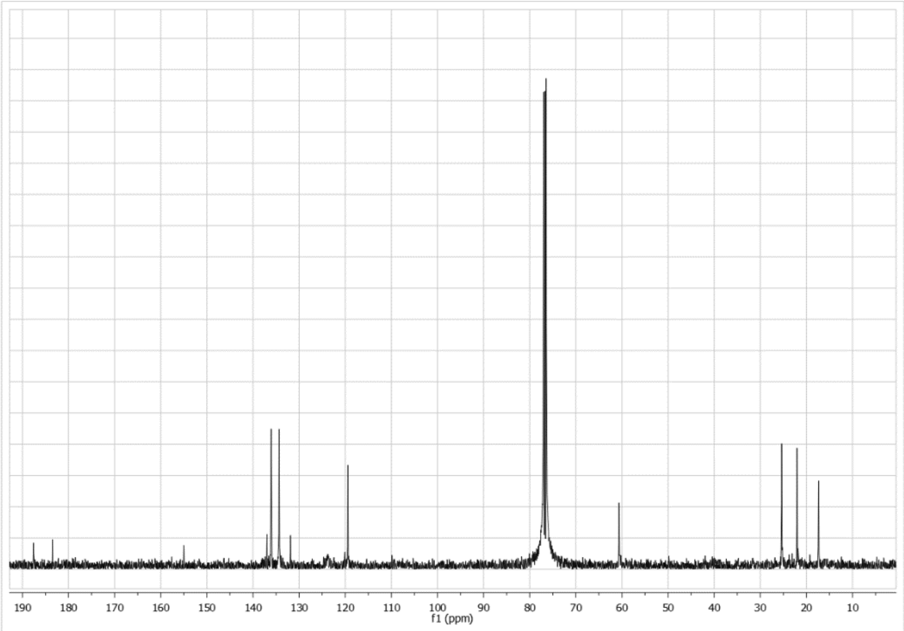

Figure S9. HRESIMS spectrum of compound **4**

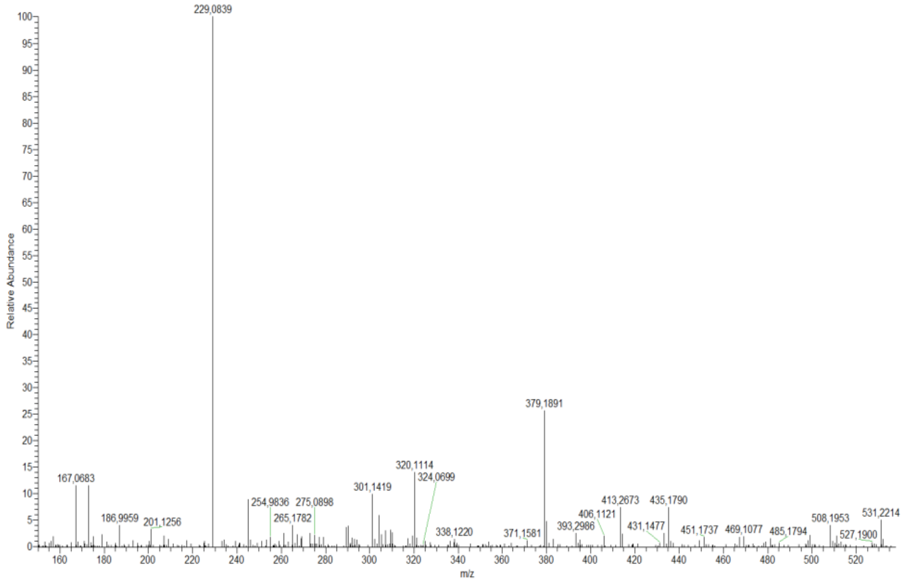

Figure S10. ^{1}H NMR spectrum in $CDCl_3$ (500 MHz) of compound **5**

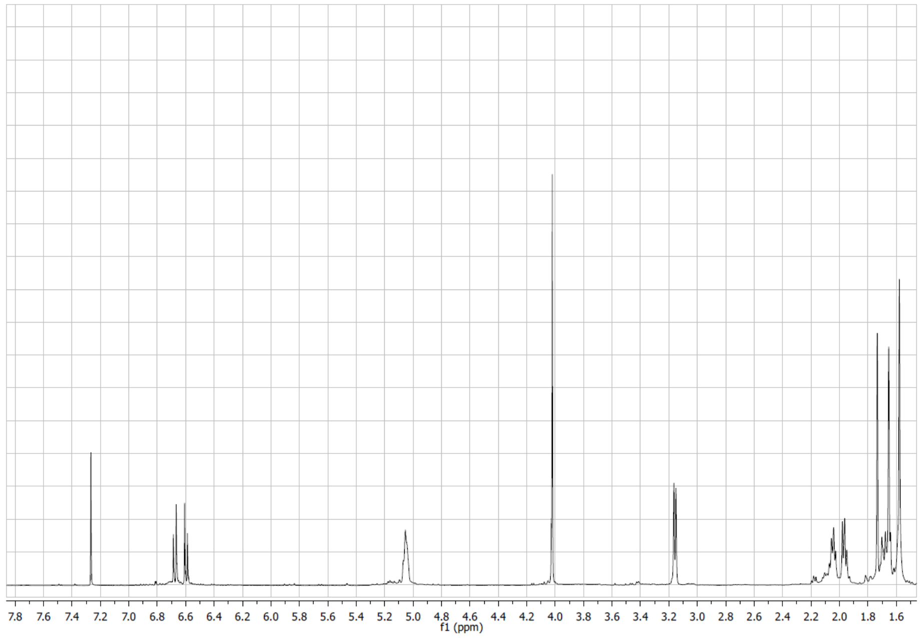

Figure S11. ^{13}C NMR spectrum in $CDCl_3$ (125 MHz) of compound **5**

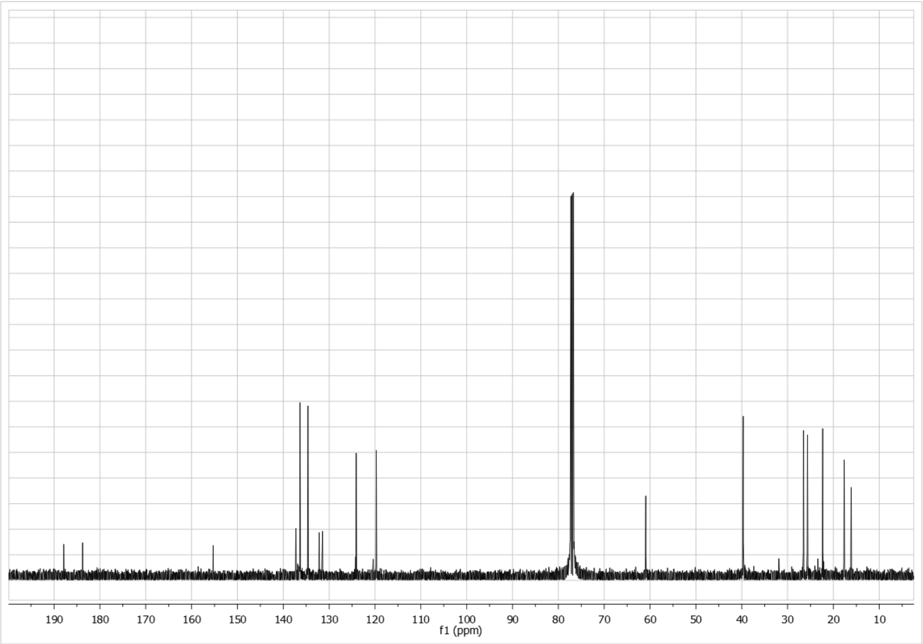

Figure S12. HRESIMS spectrum of compound **5**

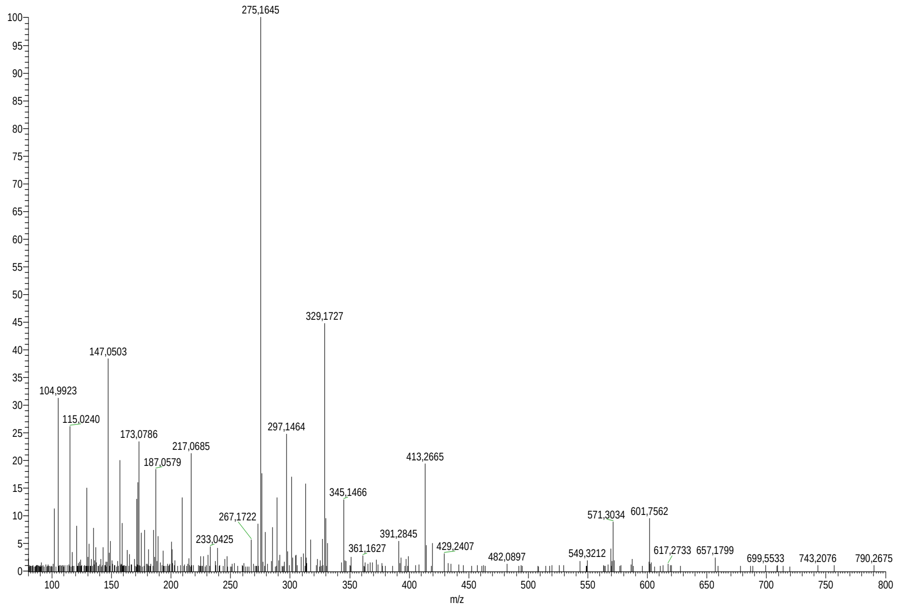

Figure S13. ^{1}H NMR spectrum in $CDCl_3$ (500 MHz) of compound **6**

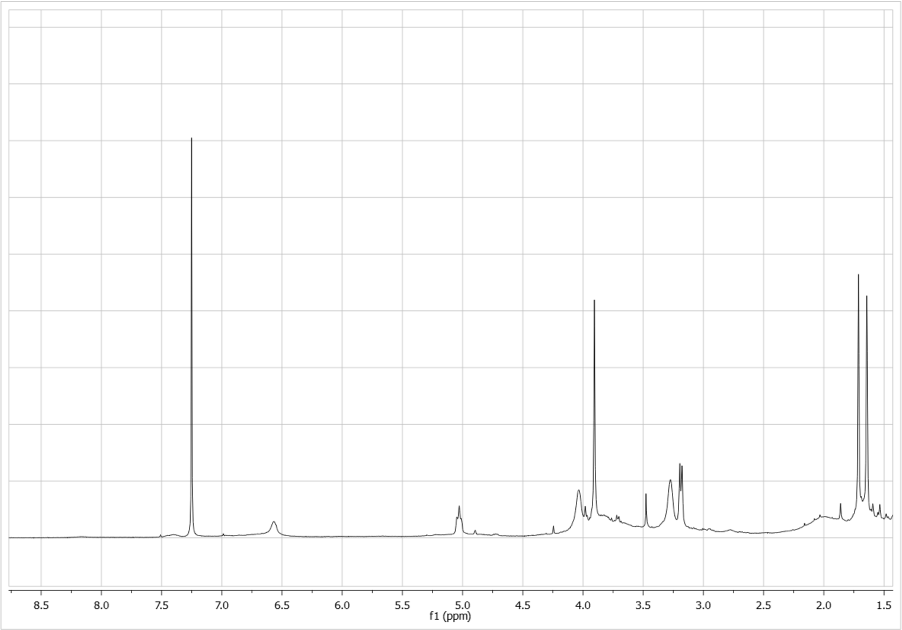

Figure S14. ^{13}C NMR spectrum in $CDCl_3$ (125 MHz) of compound **6**

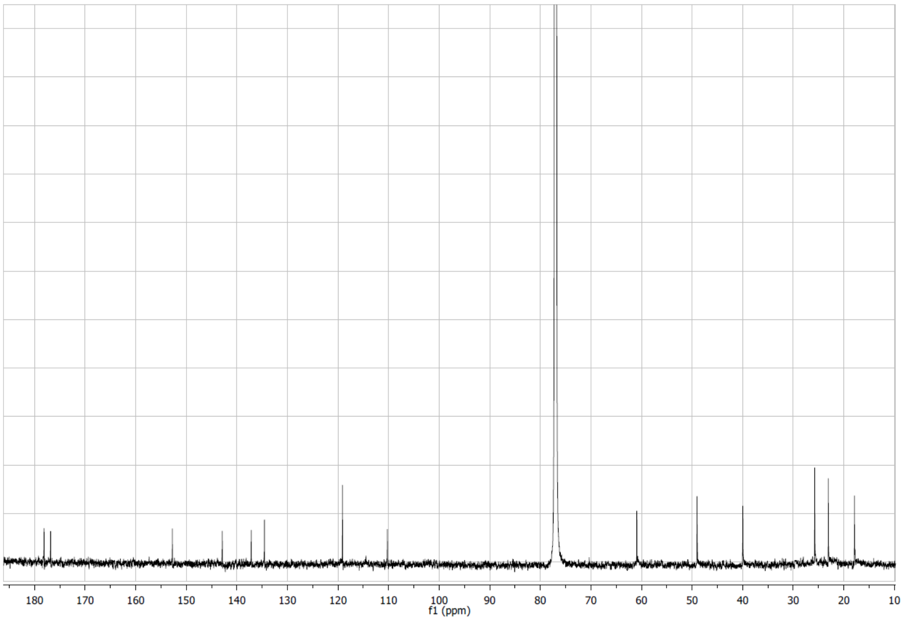

Figure S15. HMBC spectrum in $CDCl_3$ (700 MHz) of compound **6**

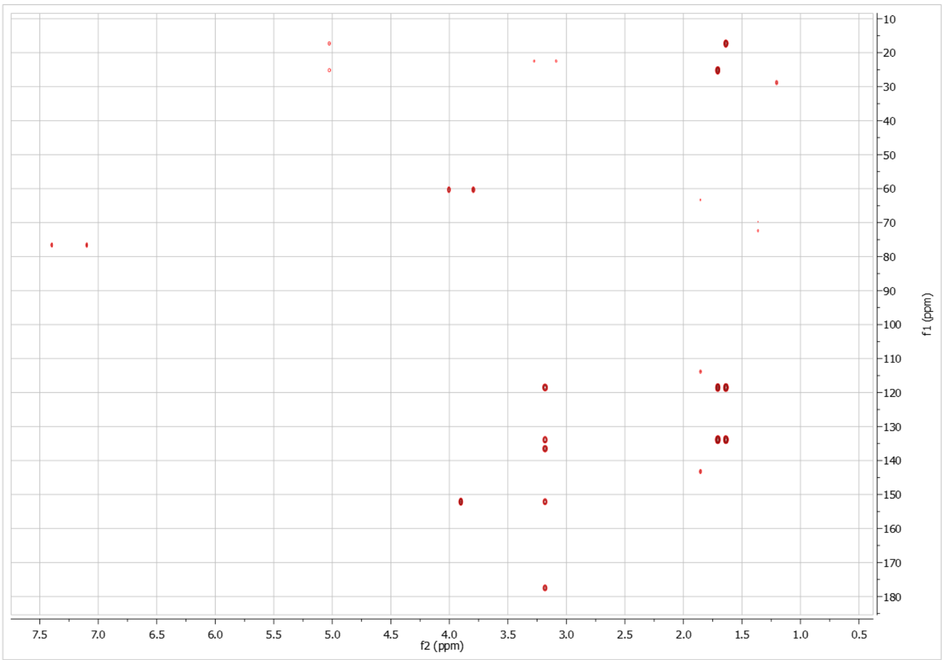

Figure S16. HRESIMS spectrum of compound **6**

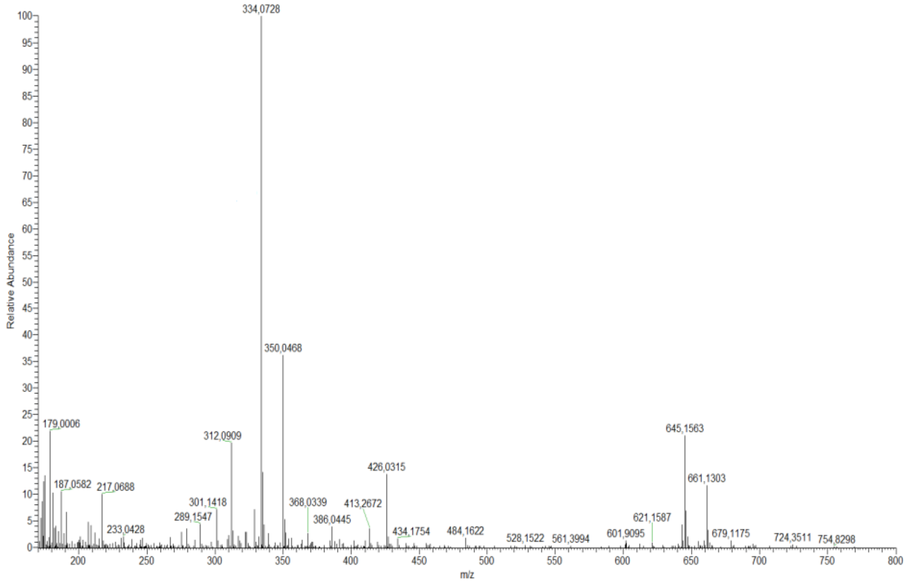

Figure S17. ^{1}H NMR spectrum in $CDCl_3$ (500 MHz) of compound **7**

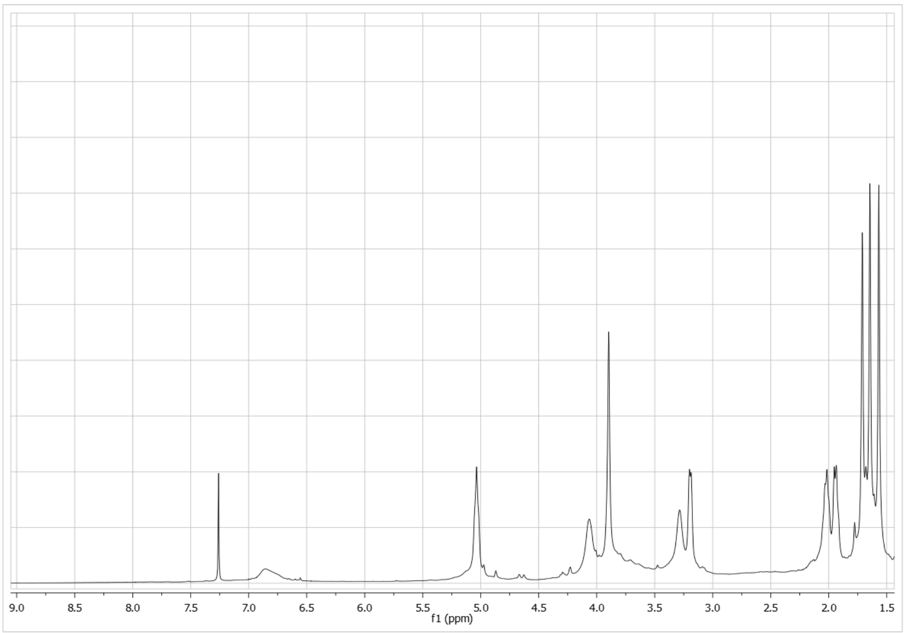

Figure S18. ^{13}C NMR spectrum in $CDCl_3$ (125 MHz) of compound **7**

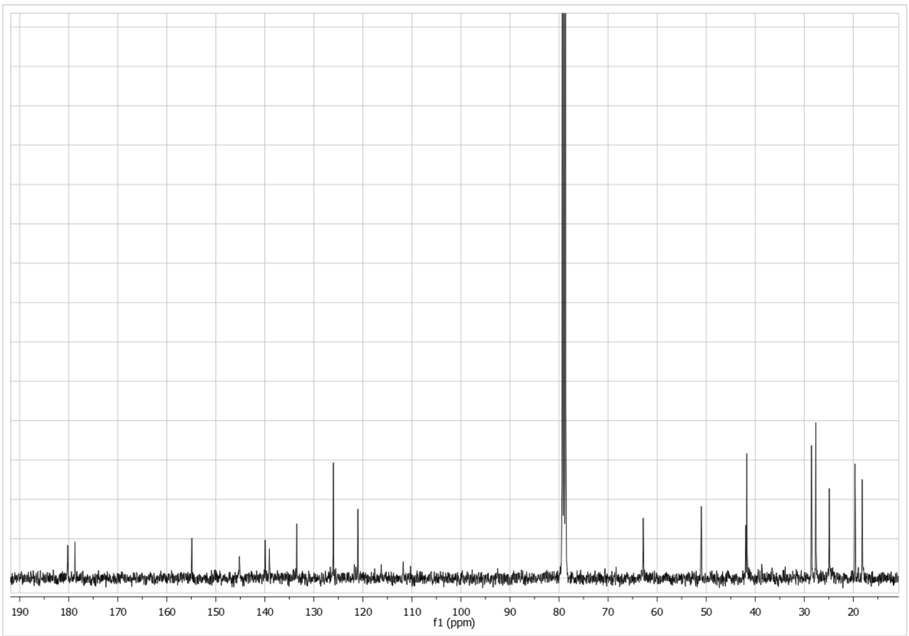

Figure S19. HMBC spectrum in $CDCl_3$ (700 MHz) of compound **7**

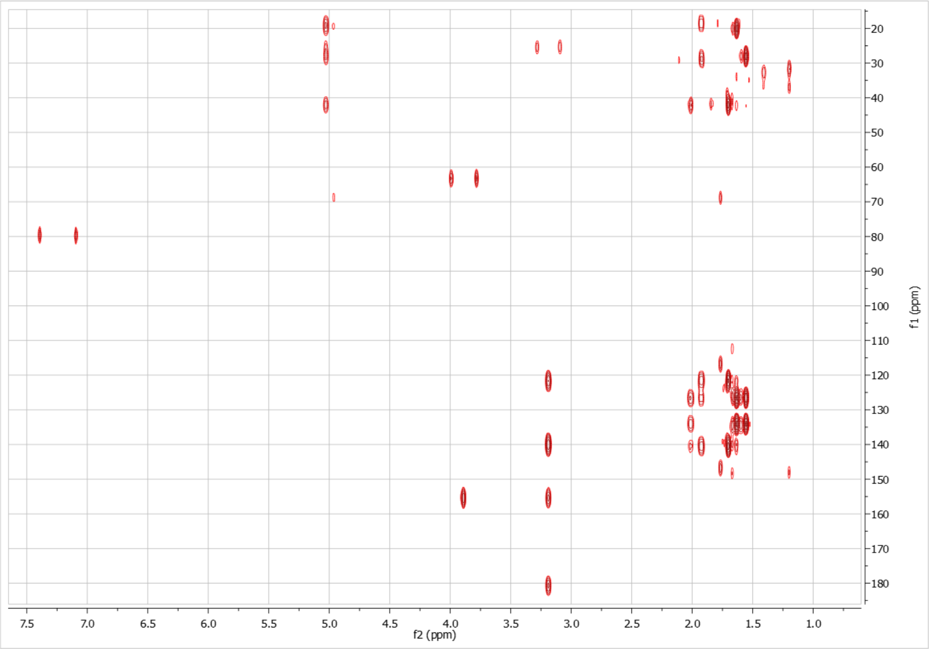

Figure S20. HRESIMS spectrum of compound **7**

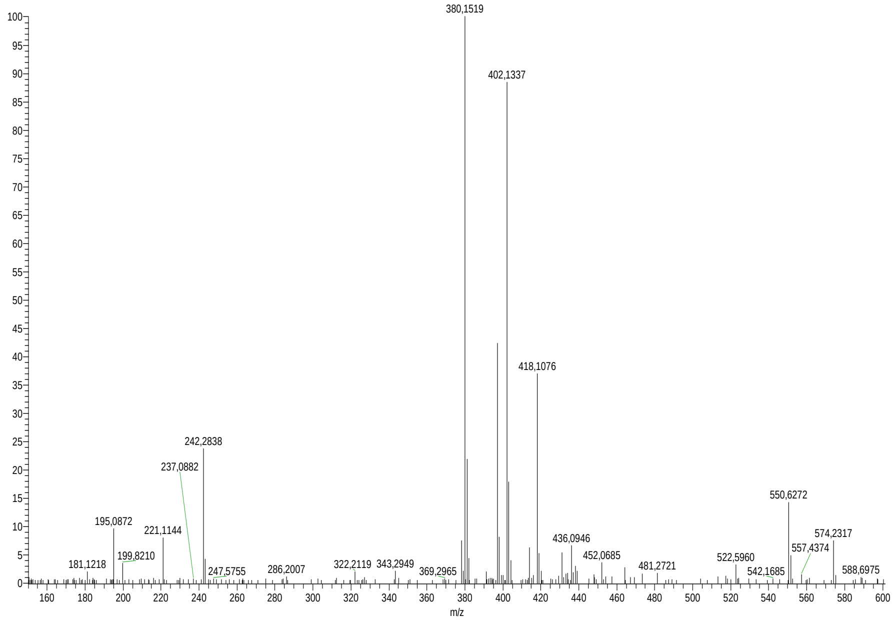

Figure S21. HPLC chromatogram of compound **4**

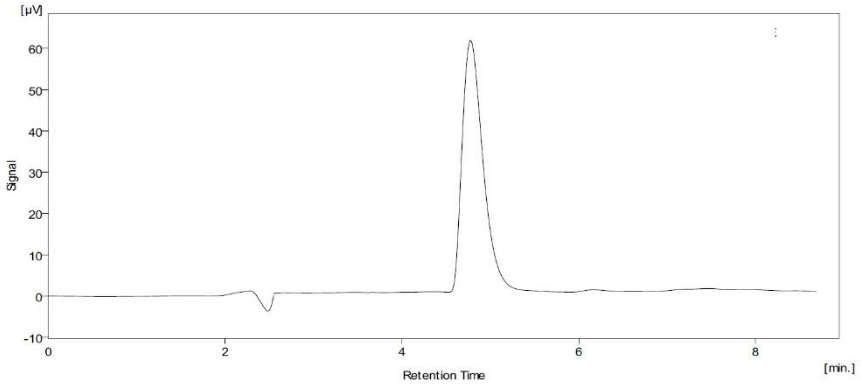

Figure S22. HPLC chromatogram of compound **5**

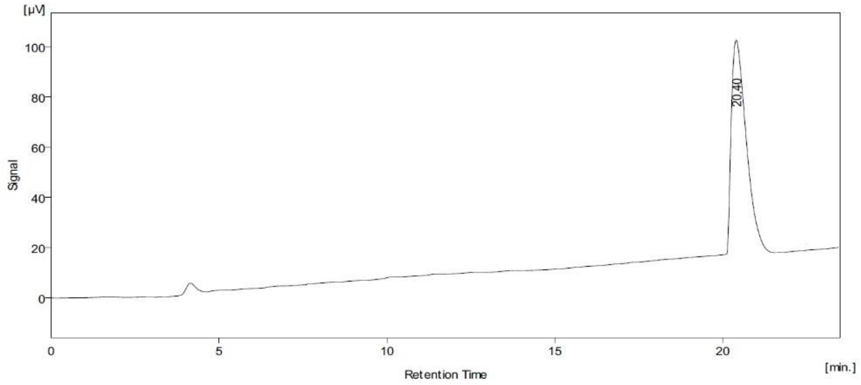

Figure S23. HPLC chromatogram of compound **6**

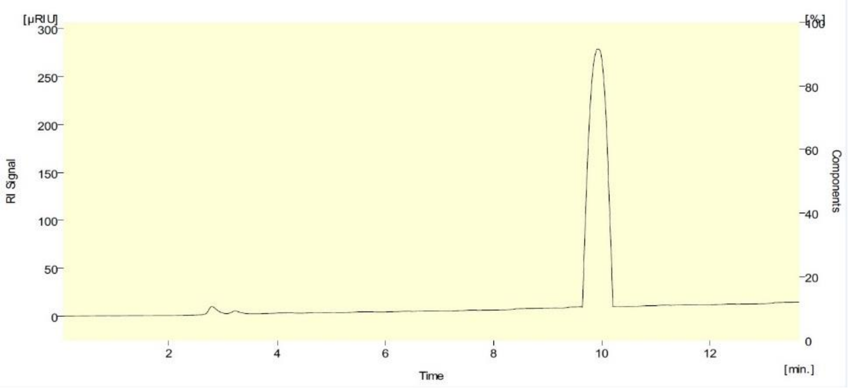

Figure S24. HPLC chromatogram of compound **7**

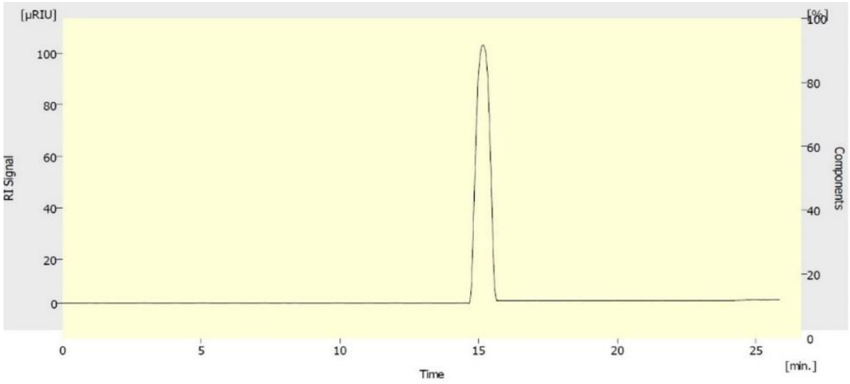

Supplement: Supplementary file 1 [file marinedrugs-17-00684-s001.pdf]
